# Psychometric Properties of the Malay Version of the Coach-Athlete Relationship Questionnaire for Coaches and Athletes

**DOI:** 10.21315/mjms2024.31.3.19

**Published:** 2024-06-27

**Authors:** Garry Kuan, Mon Redee Sut Txi, Fatin Nurfatehah Mat Salleh, Keng Yinn Wong, Huiyi Tan, Yee Cheng Kueh

**Affiliations:** 1 Exercise and Sports Science Programme, School of Health Science, Universiti Sains Malaysia, Kelantan, Malaysia; 2 Brain and Behaviour Cluster, School of Medical Sciences, Universiti Sains Malaysia, Kelantan, Malaysia; 3 Department of Coaching Science, Faculty of Sports Sciences and Coaching, Universiti Pendidikan Sultan Idris, Malaysia; 4 Faculty of Mechanical Engineering, Universiti Teknologi Malaysia, Johor, Malaysia; 5 Process Systems Engineering Centre, Universiti Teknologi Malaysia, Johor, Malaysia; 6 Faculty of Chemical and Energy Engineering, Universiti Teknologi Malaysia, Johor, Malaysia; 7 Biostatistics and Research Methodology Unit, School of Medical Sciences, Universiti Sains Malaysia, Kelantan, Malaysia

**Keywords:** coaches-athletes relationship, Malay population, athletes, psychometric properties

## Abstract

**Background:**

In the world of sports, motivation is an essential concept that can affect the sporting performance of athletes and help them accomplish their goals. The coach is regarded as an important individual with the ability to significantly influence the athlete’s motivation. To assess the impact of the coach-athlete relationship on motivation, the objective of this study was to evaluate the psychometric properties of the Malay version of the Coach-Athlete Relationship Questionnaire (CART-Q) for coaches and athletes.

**Methods:**

A cross-sectional survey was conducted among the coaches and athletes in Malaysia. Data were collected using a convenience sampling approach over a 6-month period. The study was carried out in two phases using two independent samples of coaches and athletes to assess the construct validity and internal consistency of the Malay version of the CART-Q. The CART-Q consisted of 11 items measuring three constructs: i) closeness (four items), ii) commitment (three items) and iii) complementarity (four items). In phase 1, the subjects consisted of 211 coaches (21 years old–65 years old) from both sexes and from individual and team sports, ranging from levels 1 to 5. In phase 2, the subjects consisted of 362 athletes (12 years old–39 years old), also from both sexes and from individual and team sports. The statistical analyses performed included confirmatory factor analysis (CFA) to validate the translated version scale, composite reliability (CR), average variance extracted (AVE) and internal consistency (Cronbach’s alpha).

**Results:**

In phase 1, the sample of coaches, with 190 males (90.0%) and 21 females (10.0%), had a mean age of 38.6 (SD = 8.74) years old. The major sport type was archery (19.0%). The CFA revealed adequate fit indices with all 11 items retained (root mean square error of approximation [RMSEA] = 0.059, comparative fit index [CFI] = 0.964, Tucker and Lewis Index [TLI] = 0.950, standardised root mean square residual [SRMR] = 0.037). The CR values were closeness = 0.874, commitment = 0.566 and complementarity = 0.757. The AVE values were closeness = 0.357, commitment = 0.194 and complementarity = 0.275. The Cronbach’s alpha values were closeness = 0.867, commitment = 0.553 and complementarity = 0.794. In phase 2, the sample of athletes, with 175 males (48.1%) and 189 females (51.9%), had a mean age of 20.2 (SD = 3.35) years old. The major sport type was archery (11.5%). The CFA revealed satisfactory fit indices with all 11 items retained (RMSEA = 0.092, CFI = 0.948, TLI = 0.924, SRMR =.038). The CR values were closeness = 0.893, commitment = 0.786 and complementarity = 0.949. The AVE values were closeness = 0.401, commitment = 0.253 and complementarity = 0.418. The Cronbach’s alpha values were closeness = 0.900, commitment = 0.772 and complementarity = 0.900.

**Conclusion:**

Overall, the study findings supported the conclusion that the Malay version of the CART-Q has adequate psychometric properties to assess the perceptions of coaches and athletes regarding their relationship.

## Introduction

In the context of competitive sports, social ties are crucial, impacting the professional and personal success of both athletes and coaches (1, 2). The interaction between athletes and their coaches is thought to have a significant impact on sports performance as it incorporates cognitive, behavioural and emotional components (3). According to Jowett and Poczwardowski (4), coach-athlete relationships are interactive processes in which the ideas, emotions and actions of both coach and athlete are intrinsically and interdependent. Furthermore, Jones and Turner (5) contend that this relationship is the fundamental basis of coaching and that, given the intricacy of the coaching process’ features, it is crucial for coach and athlete to be close to each other and collaborate on tasks.

According to the multidimensional model of sport leadership, interpersonal connections are traditionally studied in relation to the coach’s leadership (6). The paradigm states that the coach’s actions have a direct impact on the performance, fulfilment and motivation of a team and its members. The coach’s behaviour is, in turn, influenced by antecedents, including personal, participation and situational qualities. As a result, the majority of athletes view their coach as a role model in their lives and this coach-athlete relationship has been found to be a key factor in determining the athletes’ level of motivation (2, 7).

Athletes face numerous challenges in sports in order to succeed and achieve their objectives. Therefore, in order to succeed in their careers, they must have higher levels of motivation along with physical and psychological fortitude (8–10). The way coaches interact with athletes can have a huge impact on how motivated the athletes are to participate in sports and how much they like their coaches (10, 11). Consequently, coaches have a variety of responsibilities, including those of a mentor, teacher and leader (11, 12). A clear and effective communication between coach and athlete is supported by a positive partnership. According to Poczwardowski et al. (13), this interaction typically happens frequently during practice, competition and other contexts outside of sports, such as personal life.

For many Malaysian national athletes, one of the most important factors in their success in sports is having positive relationships with their coaches (14). However, in Malaysia, athletes and coaches come from various cultures and ethnic groups, which may have an indirect impact on the coach-athlete relationship. Coaches and athletes must reach a particular level of understanding in order to prevent misunderstandings in the racial aspect of their interaction (15). Although several studies have been conducted on the coach-athlete relationship, most of them have come from Western perspectives. Such empirical investigations have received very little attention in Malaysia (14, 16).

The coach-athlete relationship is believed to be a worldwide phenomenon that can happen anywhere that people are actively involved in sports (17, 18). Therefore, a valid instrument that evaluates the emotions, cognitions and behaviours of coaches and players is required to explore and comprehend the relationship dynamics at play between coach and athlete. Thus far, the psychometric properties of the Coach-Athlete Relationship Questionnaire (CART-Q) have been examined and verified in different populations, including Belgian coaches (19, 20), Iranian coaches (21), Chinese coaches and athletes (20), Turkish coaches and athletes (22), Polish coaches (23), Brazilian athletes (24) and Brazilian athletes and coaches (25). Some of these studies examined the psychometric properties of CART-Q from the viewpoints of both athletes and coaches, and they suggest the need for a bidirectional examination of the questionnaire (25, 26). The CART-Q has not yet been validated among Malaysian coaches and athletes. Therefore, the Malay population was the subject of two phases of the current study. Phase 1 evaluates the psychometric properties of CART-Q among coaches and phase 2 evaluates these properties among athletes.

## Methods

### Study Design and Data collection

A total of 573 participants (211 coaches and 362 athletes) were recruited for the current study, which was a cross-sectional survey, between January and July 2023 in Malaysia. The study was carried out in accordance with the Declaration of Helsinki and received approval from the Human Research Ethics Committee of Universiti Sains Malaysia, Malaysia. Coaches and athletes from all sports and competitive levels were invited to freely participate after the questionnaire had been modified and translated. The participants were notified that all research data would be kept confidential. To gather information using the CART-Q, a few days were set aside for those who were willing to participate. Following the signing of the free consent form, the data was collected in the training facilities. The study has been conducted with two different data collections: the first among coaches and the second among athletes.

### Samples Size

The minimum sample size for confirmatory factor analysis (CFA) models with seven or fewer constructs should be 300 (27). Therefore, the estimated sample size for this objective is 300. After adding the 20% dropout rate, the adjusted sample size is 375. However, it was stated that a sample size of at least 200 will offer adequate statistical power for CFA (28). Hence, a total of 362 athletes were selected and given the limited number of coaches, we considered the 211 participants to be sufficient for this study.

### Participants

Phase 1: A total of 211 coaches (aged 21 years old–65 years old), 190 males and 21 females, with mean ages of 38.6 (SD = 8.7) years old, from levels 1 (12.8%), 2 (32.7%), 3 (9.0%), 4 (31.8%) and 5 (13.7%) took part in this study. The majority of these sports include athletic (11.8%), rugby (10.0%), futsal (10.0%), hockey (10.9%) and archery (19.0%). Phase 2: A total of 362 athletes (aged 12 years old–39 years old), 175 males and 189 females, with mean ages of 20.2 (SD = 3.3) years old, from individual (47.5%) and team (52.5) sports, took part in this study. The majority of these sports include archery (11.5%), football (4.4%), handball (4.4%), hockey (7.9%), pencak silat (7.1%), pentaque (5.8%), squash (6.6%) and taekwondo (5.2%).

### Instruments

The coach-athlete relationship was evaluated using the 11-item CART-Q questionnaire (26, 29) for the constructs of closeness-affective dimension (four questions), commitment-cognitive dimension (three items) and complementarity-behavioural dimension (four items). Responses were evaluated on a 7-point Likert scale, where 1 signified ‘strongly disagree’ and 7 signified ‘strongly agree’. The arithmetic mean of each dimension’s individual items is used to calculate each dimension’s score, and higher scores denote relationships of higher quality (25).

### Questionnaire Translation

The following procedures were used to convert the English versions of the scale from earlier studies into Malay: The English version of the CART-Q was first translated into Malay while keeping the scale’s content and meaning by a bilingual researcher familiar with the scale. Second, an English-speaking native Malay speaker back translated the English version of the translated Malay text into Malay in line with previous studies (30–32). According to Brislin (33), two bilinguals can be used, with one translating from the source language to the target language and the second translating backwards from the target language to the source. Having two versions in the original language gives the researcher evidence that the version from the middle of the procedure is equivalent to the source language forms. Third, a panel of five researchers in health psychology, sport sciences, physical education and sports psychology re-examined and decided on these two versions. These versions were assessed by the panel, which compared each item to its equivalent in the original English copy. Every inconsistency was properly corrected. The panel further evaluated the items to determine whether Malaysian populations would find them culturally appropriate (30, 32).

### Statistical Analysis

Only the questionnaires with complete responses were included for the analysis after the data were pre-screened to look for incorrect data entry and missing values. The first hypothesized models were examined using a CFA with Mplus 8. Because of its ability to execute CFA with non-normal data distributions and because it offers reliable estimates with standard errors, including a mean-adjusted chi-square statistic, the robust MLR estimator was chosen for this analysis (34).

The initial measurement models for the coaches and athletes were tested using CFA. The standardised factor loading (FL) of 0.40 and higher was applied as a cut-off to establish sufficient FL for all the items, and as such, it was employed as a criterion to retain or remove an item (35, 36). For the coaches sample, the recommended fit indices for a sample size less than 250 with less than 12 items were: root mean square error of approximation (RMSEA) with a desired value of less than 0.08; standardised root mean square residual (SRMR) with a desired value of less than 0.08; and comparative fit index (CFI) or Tucker and Lewis Index (TLI) with the desired values of more than 0.97 (27). For the athlete sample, the recommended fit indices for a sample size of more than 250 with less than 12 items were: RMSEA less than 0.07, SRMR less than 0.08, and CFI or TLI with a value of 0.95 or greater (27). To improve the model fit indices, model re-specification was done by adding residual covariances of items within the same factor using the CFA modification index. After the researchers decided that the models provided adequate theoretical meaning, the models were respecified. Additionally, the composite reliability (CR), Cronbach’s alpha and average variance extracted (AVE) were computed in order to assess the convergent validity of the Malay version of CART-Q in the present study. Cronbach’s alpha remains the most frequently cited coefficient for reliability in the literature (26). However, Cronbach’s alpha may considerably underestimate reliability and CR offers a more accurate estimate when residual covariances are taken into account in the model (37). In this study, because residual covariances were added for both the coach and athlete models and previous studies (25, 26) reported both the CR and the Cronbach’s alpha, we therefore reported both. Discriminant validity was examined by estimating the correlation coefficient between the factors. Fornell and Larcker (38) state that for discriminant validity to be supported, the AVE of the constructs must be greater than the shared variance (i.e. the square of the correlation between the constructs). In Mplus 8.0, CR was computed using Raykov’s approach (37). For CR and AVE, the cut-off values were equal to or greater than 0.60 and 0.50, respectively (38).

## Results

The general characteristics of the study respondents’ for the coaches and athletes samples are shown in Table 1. There were 211 coaches, with males making up 90.0% and females making up 10.0%. More than half of the coaches (57.3%) belong to the team sports category. In contrast, the athlete sample included 362 respondents with a mean age of 20.2 (SD = 3.35) years old, with males making up 48.1% and females making up 51.9%. More than half of the athletes (52.5%) belong to the team sports category.

### Psychometric Properties of the Coach-Athlete Relationship Questionnaire for Coaches

**Figure 1:**
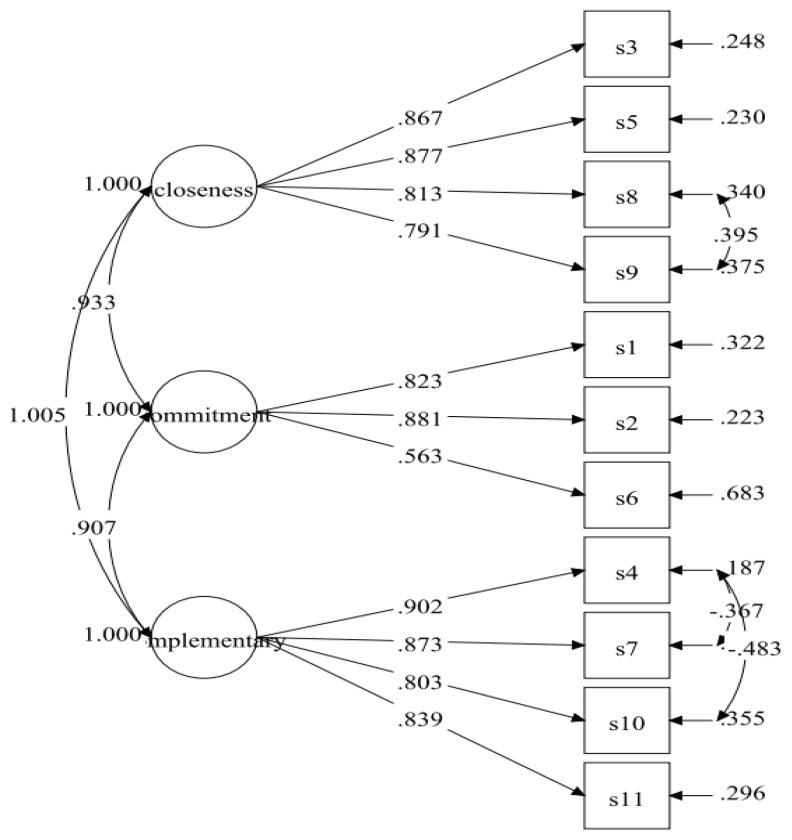
CART-Q measurement model for athletes (Model-2)

**Figure 2:**
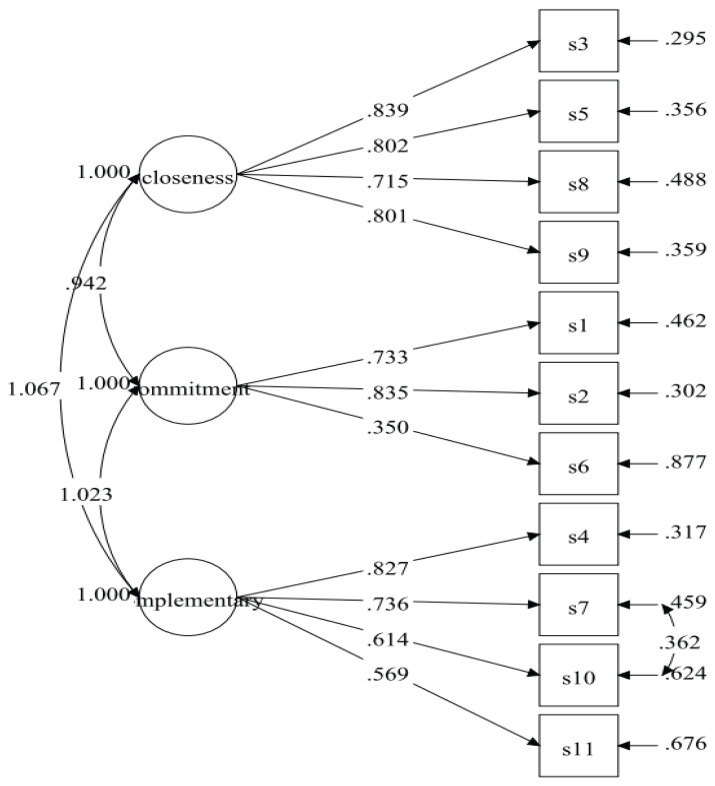
CART-Q measurement model for coaches (Model-2)

The initial specified measurement model of the CART-Q for coaches was identical to the measurement model used in the original version of the instrument (26, 29). The results of the initial specified measurement model (Model-1) displayed poor fit indices (Table 2). The model fit indices were improved after adding covariances between residuals’ items between S7 and S10 for the complementarity factor (Figure 1). The fit indices of the respecified model (Model-2) were desirable (Table 2) with all the items retained. The result for Model-2 showed standardised item loading ranging from 0.350 to 0.839, which were considered moderate to very good (Table 3, Figure 2).

### Psychometric Properties of the Coach-Athlete Relationship Questionnaire for Athletes

The initial specified measurement model of the CART-Q for athletes was also identical to the measurement model used in the original version of the instrument (26, 29). The results of the initial specified measurement model (Model-1) displayed poor fit indices (Table 4). The model fit indices were improved after adding covariances between residuals’ items between S8 and S9 for the closeness factor; S4 and S7; and S4 and S10 for the complementarity factor. The fit indices of the respecified model (Model-2) were desirable (Table 4), with all the items retained. The result for Model-2 showed standardised item loading ranging from 0.563 to 0.902, which were considered moderate to very good (Table 5).

### Composite Reliability and Average Variance Extracted

Table 6 presents the CR, AVE, factor correlation and squared correlation of the final Malay version of the CART-Q for coaches. The CR values were: closeness = 0.874, commitment = 0.566 and complementarity = 0.757. The AVE values were: closeness = 0.357, commitment = 0.194 and complementarity = 0.275.

Table 7 presents the CR, AVE, factor correlation and squared correlation of the final Malay version of the CART-Q for athletes. The CR values were: closeness = 0.893, commitment = 0.786 and complementarity = 0.949. The AVE values were: closeness = 0.401; commitment = 0.253 and complementarity = 0.418.

### Internal Consistency

For the coaches CART-Q, the Cronbach’s alpha values were: closeness = 0.867; commitment = 0.553; and complementarity = 0.794. For the athletes CART-Q, the Cronbach’s alpha values were: closeness = 0.900; commitment = 0.772; and complementarity = 0.900.

## Discussion

The objectives of this study were to perform a translation, assess the internal consistency and investigate the construct validity of the CART-Q’s coach and athlete versions in Malay. The scales demonstrated satisfactory results for construct validity and internal consistency, showing the reliability and validity of the Malay version for evaluating the coach-athlete relationship from the viewpoints of both coach and athlete. This is the first study to conduct such an analysis in the context of Malaysian sports, emphasising both its contribution to scientific understanding in this field and the applicability of the scale. As a result, this study can aid in the quest for the best possible team performance in sports during practice and competition.

Consistent with the validation tests carried out in other cultures, the instrument’s original 11-item structure with three factors (closeness, commitment and complementarity) was retained (19, 20, 25, 26). These findings suggest that the CART-Q evaluates the overall nature of the coach-athlete relationship, which includes the emotions, ideas and actions of these participants in the sport (20, 25). The CR analysis, which was seldom reported in prior validation research from other countries, is further progress made by the current study.

The three-factor model comprising the 11 items in the current study’s CFA analysis for the CART-Q for coaches revealed sufficient fit indices. This finding confirms earlier findings and presents researchers with confidence in the CART-Q’s three-dimensional psychometric properties (20, 25). All of the factors, with the exception of item 6 (the commitment factor), have FL above 0.40; however, it was decided to keep this item in the model. Such evidence was also found in the earlier studies, where items 4 and 11, from the complementarity factor, had low FL (20, 25). This finding supports the need for continuous psychometric analyses of this measure across cultural contexts, as suggested by previous research.

The current study indicated that the fit indices values for CFI and TLI for the CART-Q for athletes were lower than recommended, along with high RMSEA value. All of the FL for the 11 items, however, were higher than 0.40. Lower fit indices and a higher RMSEA value have been reported in previous validation studies of the CART-Q for athletes and coaches (25, 26). This result is also consistent with Woolliams et al. (18) earlier research, which stated that a unidimensional structure yielded more robustness than a multidimensional model. Consequently, a unidimensional factor solution may explain the model more accurately than was previously claimed.

In addition, the Cronbach’s alpha obtained for the CART-Q for both coaches and athletes was higher than the minimum recommended value of 0.70 (37), with the exception of the commitment factor for the coach sample. The fact that all item-total correlation coefficients were higher than 0.30, however, shows that the items significantly differentiated the individuals based on the attribute being measured (35). All of the CR values were higher than the minimum recommended levels (> 0.60) except commitment factor for coach sample (0.566), demonstrating the internal consistency of this version of the CART-Q for coaches and athletes, which is consistent with earlier studies (19, 20, 25, 26). None of the AVE values from the samples of coaches and athletes were greater than the recommended value (> 0.50). However, given that these factors are evaluating related constructs, such outcomes were always anticipated (26, 31, 37). Also, the squared correlation coefficients were all less than the AVE values of its factors, except for the commitment factor for the coach’s sample. These results showed that most of the factors had sufficient discriminatory validity (38).

Finally, in this study, the MI recommended adding three pairs of covariances between residuals’ items, between S8 and S9 (in the closeness factor), and, between S4 and S7 and S4 and S10 (in the complementarity factor) for the CART-Q athletes, as well as one pair of covariances between residuals’ items S7 and S10 in the complementarity factor for CART-Q coaches. In previous studies that examined the psychometric properties of the CART-Q’s athlete version, these improvements were also performed to achieve better model fit (19, 25). Such outcomes can be connected to the intercultural aspects of interpersonal relationships (25). However, in social psychology research, these covariances should be taken into account when they provide a significant improvement to the model (39, 40).

There are some limitations related to this study. Firstly, using a self-reported survey could lead to response bias and lower the accuracy of the data obtained. To manage this complexity, all the participants received assurances regarding the privacy of their data and were encouraged to complete the surveys honestly, answering all the questions based on their genuine perceptions. Secondly, the sample size of this study posed a challenge for the application of CFA. Although the sample size per free parameter ratio was reached, the single environment in which the data were obtained may prevent generalisation of the results. Thirdly, the study’s sample of coaches was smaller than its sample of athletes because there are generally fewer coaches than athletes. Future research is required to determine how participants read and interpret the items. For participants to better comprehend the meaning of each item and reduce the likelihood of any misinterpretation, a guide with an explanation of the questions may be necessary.

## Conclusion

The results of this study showed that the construct validity and internal consistency of the Malay version of the CART-Q for coaches and athletes were satisfactory. As a result, the study demonstrated that it is possible to evaluate the coach-athlete relationship from the viewpoints of both coaches and athletes using the Malay versioned CART-Q. In this regard, the findings are pertinent to the training of both coaches and athletes, as strong interpersonal relationships in sports can benefit the performance and overall well-being of both parties. The scale and knowledge that have been discussed will prove useful to sport psychologists as they design research projects and intervention programmes aimed at promoting healthy social interactions in athletic competitions. Given that both the athlete and coach versions of this instrument have been validated and are currently available for future research and practical use, a bidirectional assessment of the coach-athlete relationship is thus made possible in the national Malaysian context.

## Figures and Tables

**Table 1:** General characteristics of the study respondents

Variables	Coaches (*N* = 211)		Athletes (*N* = 362)	

	Mean (SD)	*n* (%)	Mean (SD)	*n* (%)
Age (years old)	38.6 (8.74)		20.2 (3.35)	
Gender				
Male		190 (90.0)		175 (48.1)
Female		21 (10.0)		189 (51.9)
Sport category				
Individual		90 (42.7)		173 (47.5)
Team		121 (57.3)		191 (52.5)

**Table 2:** Summary of the CART-Q fit indices for coaches

Path model	RMSEA (90% CI)	CFI	TLI	SRMR
Model-1	0.072 (0.050, 0.093)	0.945	0.926	0.041
Model-2^a^	0.059 (0.034, 0.082)	0.964	0.950	0.037

Note:

a Model-2 with correlated items residual; S7 with S10

**Table 3:** Item’s descriptive statistics and standardised FL of the coaches (*N* = 211)

Item content	Mean	SD	FL
Closeness			
S3	6.33	0.896	0.839
S5	6.33	0.825	0.802
S8	6.56	0.669	0.715
S9	6.56	0.762	0.801
Commitment			
S1	6.12	0.991	0.733
S2	6.24	0.886	0.835
S6	5.27	1.718	0.350
Complementarity			
S4	6.40	0.739	0.827
S7	6.58	0.747	0.736
S10	6.73	0.540	0.614
S11	6.36	0.841	0.569

**Table 4:** Summary of the CART-Q fit indices for athletes

Path model	RMSEA (90% CI)	CFI	TLI	SRMR
Model-1	0.109 (0.095, 0.124)	0.921	0.893	0.043
Model-2^a^	0.092 (0.077, 0.107)	0.948	0.924	0.038

Note:

a Model-2 with correlated items residual; S8 with S9, S4 with S7, S4 with S10

**Table 5:** Item’s descriptive statistics and standardised FL of the athletes (*N* = 362)

Item content	Mean	SD	FL
Closeness			
S3	6.01	1.268	0.867
S5	6.17	1.202	0.877
S8	6.58	0.901	0.813
S9	6.50	0.955	0.791
Commitment			
S1	5.62	1.386	0.823
S2	5.96	1.247	0.881
S6	5.12	1.573	0.563
Complementarity			
S4	5.98	1.242	0.902
S7	6.10	1.161	0.873
S10	6.43	0.990	0.803
S11	6.21	1.136	0.839

**Table 6:** CR, AVE, factor correlation and squared correlation of the final Malay version CART-Q for coaches

Construct	CR (95% CI)	AVE	1	2	3	*r* *^2^*
Closeness	0.874 (0.841, 0.906)	0.357	1	0.512	0.488	0.262
Commitment	0.566 (0.457, 0.675)	0.194		1	0.452	0.204
Complementarity	0.757 (0.663, 0.851)	0.257			1	0.238

**Table 7:** CR, AVE, factor correlation and squared correlation of the final Malay version CART-Q for athletes

Construct	CR (95% CI)	AVE	1	2	3	*r* *^2^*
Closeness	0.893 (0.860, 0.927)	0.401	1	0.182	0.240	0.033
Commitment	0.786 (0.730, 0.841)	0.253		1	0.164	0.027
Complementarity	0.949 (0.927, 0.971)	0.418			1	0.058
